# Thermal damage thresholds for multiple-pulse porcine skin laser exposures at 1070 nm

**DOI:** 10.1117/1.JBO.25.3.035001

**Published:** 2019-09-05

**Authors:** Michael P. DeLisi, Morgan S. Schmidt, Aaron F. Hoffman, Amanda M. Peterson, Gary D. Noojin, Aurora D. Shingledecker, Adam R. Boretsky, David J. Stolarski, Semih S. Kumru, Robert J. Thomas

**Affiliations:** a SAIC, JBSA Fort Sam Houston, Texas, United States; b 711th Human Performance Wing, Airman Systems Directorate, Bioeffects Division, Optical Radiation Bioeffects Branch, JBSA Fort Sam Houston, Texas, United States

**Keywords:** multiple pulse, laser damage, minimum visible lesion, skin injury, near-infrared lasers

## Abstract

As solid-state laser technology continues to mature, high-energy lasers operating in the near-infrared (NIR) band have seen increased utilization in manufacturing, medical, and military applications. Formulations of maximum permissible exposure limits establish guidelines for the safe use of these systems for a given set of laser parameters, based on past experimental and analytical studies of exposure thresholds causing injury to the skin and eyes. The purpose of our study is to characterize the skin response to multiple-pulsed laser exposures at the NIR wavelength of 1070 nm, at a constant beam diameter of 1 cm, using anesthetized Yucatan mini-pig subjects. Our study explores three constant total laser-on times of 0.01, 0.1, and 10 s as single- and multiple-pulse sequences. Exposures consisting of 10, 30, and 100 pulses have identical individual pulse durations but different duty cycles in order to include variable degrees of thermal additivity. A plurality of three observers quantifies skin damage with the minimally visible lesion metric, judged at the 1- and 24-h intervals postexposure. Calculation of the median effective dose (${\mathrm{ED}}_{50}$) provides injury thresholds for all exposure conditions, based on varying laser power across subjects. The results of this study will provide a quantitative basis for the incorporation of multiple-pulsed laser exposure into standards and augment data contained in the existing ${\mathrm{ED}}_{50}$ database.

## 1 Introduction

A continually expanding array of laser technology requires vigilant monitoring of the mechanisms of biological exposure damage and assuring adequacy of the data informing the standards of safe use. Solid-state lasers comprise one category that has undergone substantial recent development in high-powered implementations. The safety hazard to skin is particularly relevant in high-energy laser applications, where systems are capable of delivering enough energy to cause severe burn injuries. Several of these lasers operate in the near-infrared (NIR) region of the electromagnetic spectrum, which includes the wavelength range of 800 to 2500 nm. Experimental studies of skin injury thresholds are specific to the wavelength of the laser. These thresholds provide the basis for the formulation of maximum permissible exposure limits in the accepted laser safety standards, such as the American National Standards Institute (ANSI) Z136.1-2014^1^ and the International Electrotechnical Commission (IEC) 60825-1.^2^ The continual development of these threshold databases ensures that current and emerging systems have appropriately validated exposure limits, based not just on theoretically predicted mechanisms but also experimental observations.

Rockwell and Goldman^3^ conducted one of the first extensive studies on laser skin damage for low- and high-pigmented human skin, across various visible and infrared wavelengths. Their findings include minimum visible lesion (MVL) thresholds for exposure durations of 75 ns and 1 s at 1060 nm. Subsequent studies also explored the 1060-nm wavelength for both human and porcine subjects, for 1-s^4^^,^^5^ and 0.2- to 0.3-s^6^^,^^7^ exposures. A recent study by Vincelette et al.^8^ determined the contribution of beam diameter to porcine skin MVL thresholds for 1070-nm laser exposure durations ranging from 10 ms to 10 s. DeLisi et al.^9^ further investigated skin injury response to 1070-nm lasers but at suprathreshold endpoints for 3- and 100-ms exposures in excised porcine tissue. Cain et al.^10^ and Montes de Oca et al.^11^ presented porcine skin MVL thresholds for light near 1315 nm and pulse durations of 0.35 ms, whereas Oliver et al.^12^ provided thresholds at 1319 nm for exposure durations of 0.25, 1, 2.5, and 10 s. Several porcine skin MVL threshold studies for 1540-nm laser are available, including for 30-ns,^13^^,^^14^ 0.6-,^13^ 0.8-,^15^ and 60-ms pulses.^16^ Chen et al.^17^^–^^19^ extensively studied the effects of 2000-nm laser porcine skin damage. Exploration of this wavelength region is supplemented by Oliver et al.^20^ in a study of 1940-nm laser thresholds, which employed exposure durations from 10 ms to 10 s and beam diameters of 4.8 to 18 mm. Chen et al.^21^ is also responsible for one of the only studies on 1214-nm porcine skin damage thresholds. Many of the porcine skin studies employed the Yucatan mini-pig, which is an established model for human skin NIR exposure damage due to its morphological and physiological similarities to human skin.^22^

Most of the aforementioned studies are for singular instances of uninterrupted laser radiation, such as a short discrete pulse or a longer continuous-wave (CW) exposure. However, many laser systems can deliver multiple pulses in rapid succession. Any damage assessment of such an exposure must take into account the cumulative effect of these pulses.

There has been extensive research into multiple-pulse MVL thresholds in the eye using number of pulses and pulse repetition frequency (PRF) as variables, for the infrared^23^^–^^26^ and visible^27^^–^^31^ wavelength regions. Clark et al.^32^ provided a theoretical analysis of multiple-pulse thermal damage thresholds to the retina based on the Arrhenius integral model, for both visible and NIR wavelengths. Many of these studies focus on the photomechanical damage mechanism that is characteristic of very “short” ($<10^{-6}\;s$) pulses,^33^^–^^35^ which is of particular relevance in the eye due to the presence of retinal pigment epithelium, the limiting factor of blink response, and the greater sensitivity of vision to tissue damage-related dysfunction. However, other systems are capable of emission modulation to effectively deliver a multiple-pulsed output of “long” pulses ($>10^{-6}\;s$), where the tissue damage is primarily photothermal and dependent on the factors influencing thermal additivity between pulses.

With regards to skin exposure, Milanič et al.^36^ presented a framework for simulating multiple-pulse laser treatment of port wine stains at 532 nm, at an individual pulse duration of 1 ms with various numbers of pulses and PRFs. In one of the few experimental studies available, Majaron et al.^37^ investigated multiple 550-μs pulses of Er:YAG laser exposure for laser skin resurfacing, finding that dermal collagen coagulation was possible with minimal epidermal ablation under cryogen spray cooling conditions. Little experimental work is available regarding multiple-pulse effects on the skin in the NIR region, particularly with regard to MVL thresholds.

An additional point of interest in this study was to determine the contribution of hair follicles on skin damage during 1070-nm laser exposure. Nd:YAG lasers emitting at 1064 nm are well-established tools in the technique of laser hair removal.^38^^,^^39^ This observation is due to a significant disparity between the optical absorption coefficients of hair ($10^{-1}\;{\mathrm{cm}}^{-1}$) and skin tissues ($10^{-1}\;{\mathrm{cm}}^{-1}$) for this wavelength region.^40^^–^^44^ Vincelette et al.^8^ reported that high-energy exposures of porcine skin at 1070 nm often resulted in distinctly observable incineration events, with hair follicles catching fire and burning away. High-speed thermal imagery from that study reveals the drastically higher temperatures of hair follicles when compared to the baseline tissue within the beam. As a result, the hair follicles behave as small but intense transient heat sources during laser exposure, possibly contributing to additional thermal injury of the skin.

In order to address part of this data gap and as a continuation of the work started by Vincelette et al.,^8^ we performed a series of *in vivo* experiments using Yucatan mini-pigs to determine the MVL limits for 1070-nm multiple-pulse skin exposure to relatively “long” pulses (100  μs to 10 s). Given the average power limitations of the lasers and the recognition that the primary damage mechanism would be photothermal, we subdivided pulse train parameters into “total on-times” (TOTs) of 0.01, 0.1, and 10 s and differentiated them with duty cycle as opposed to the PRF. The individual pulse durations ranged from 100 ms to 10 s, with the number of pulses and pulse spacing (duty cycle) varied to examine the impact of the thermal additivity. Additionally, the use of both waxed and shaved subjects for a subset of exposures allowed assessment of the damaging effect of hair follicles.

## 2 Methods

### 2.1 Laser Optics and Camera Systems Configuration

**Fig. 1:**
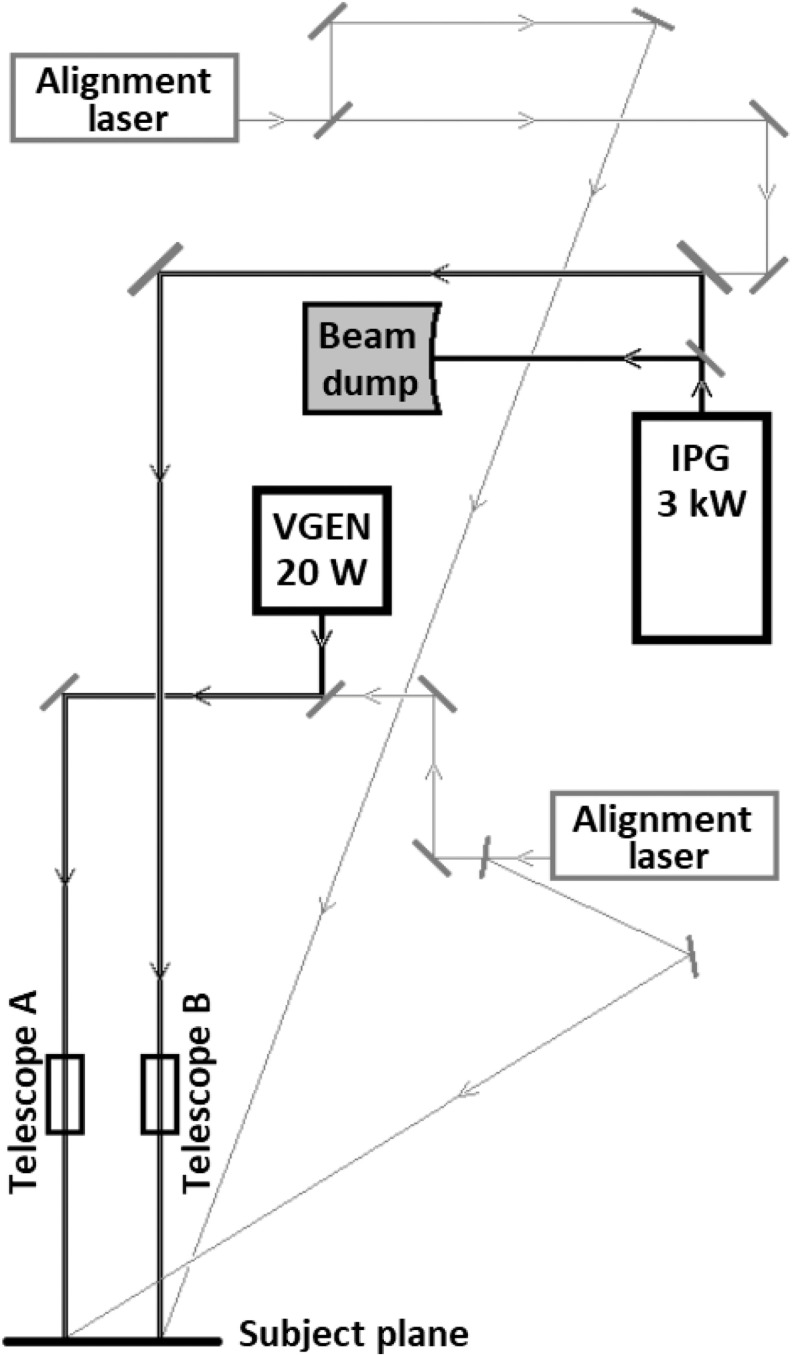
Experimental setup used for 1070-nm skin exposures.

We utilized two different Ytterbium (Yb) fiber 1070-nm laser systems for this study, seen in Fig. 1. The shorter laser exposures (0.01- and 0.1-s TOT) employed an IPG Photonics (Oxford, Massachusetts) model YLR-3000 laser with a maximum CW power of 3 kW, whereas the longer exposures (10-s TOT) utilized a 20-W Spectra-Physics (Santa Clara, California) VGEN model VCFL-20000. The output of both lasers was approximately Gaussian in spatial irradiance distribution. Beam telescopes ensured that the beam diameter for both systems was ∼1  cm ($1/e^{2}$) at the target plane, which was large enough to impose a highly thermally confined state within the beam.

An electronic control within the laser head adjusted power at the target via preset programming. The power on target was determined by calibrating the power difference between the laser setting and the power delivered on the subject plane as measured by a Coherent (Santa Clara, California) thermopile detector. The average laser power determined the selection of the detector (Coherent models PM10, PM150-50C, and PM5K). Integration of two alignment lasers into the optical setup, one co-aligned and one cross-aligned, allowed for consistent identification of the plane of exposure.

A TC-1122 camera connected to a LBA-PC frame grabber (Ophir-Spiricon, North Logan, Utah) provided measurements of the beam size on target by imaging 1% front reflectance by an optical wedge of the beam onto a scattering plate with an optical diffuse material (Gigahertz-Optik, Amesbury, Massachusetts) at a coincident plane to the exposure site. Thus beam diameters measurements occurred at the same distance from the laser output aperture as the exposure plane. We measured the beam diameters of both laser systems a total of five times throughout the experiment. The YLR-3000 laser had a mean diameter of 1.04 cm and standard deviation of 0.044 cm, whereas the VGEN 20-W laser had a mean diameter of 0.973 cm with a standard deviation of 0.020 cm, resulting in a 6.66% difference between the two beam diameters.

In order to record intraexposure tissue temperature rise, we mounted a SC6800 (IR) InSb high-speed thermal camera (FLIR Systems, Boston, Massachusetts) on a custom translation stage and focused it on the expected exposure plane. This system is sensitive to radiation in the 3- to 5-μm wavelength thermal emission band, with a JANOS Technology (Keene, New Hampshire) 100-mm thermal imaging lens. This camera sensitivity was out-of-band for the lasers under test so that it could detect intraexposure surface temperature changes without target backscatter distortions. The thermal camera and laser control electronics operated in synchronization to ensure a thermal data collection initiation at 40 ms prior to laser activation. The camera collected data at 1440 or 200 Hz for exposure durations less or greater than 10 s, respectively. The total number of frames collected for any given exposure allowed for several seconds of capture beyond the completion of each laser exposure in order to measure the trajectory of thermal decay of the laser-heated tissue. A technician periodically calibrated the camera using a M345 blackbody (Mikron, Oakland, New Jersey) with a 15°C to 120°C range. A CCD color video camera in co-focus with the thermal camera provided real-time target position verification.

### 2.2 Animal Care

The animals involved in this study were procured, maintained, and used in accordance with the Federal Animal Welfare Act, “Guide for the Care and Use of Laboratory Animals,” prepared by the Institute of Laboratory Animal Resources National Research Council, and Army Regulation 40-33 Secnavinst 3900.38C AFMAN 40-401(1) DARPAINST 18 USUHSINST 3203 “The Care and Use of Laboratory Animals in DOD Programs.” The Air Force Research Laboratory at JBSA-Fort Sam Houston, Texas has been fully accredited by the Association for Assessment and Accreditation of Laboratory Animal Care, International since 1967.

Yucatan mini-pigs went without food for 12 h prior to anesthetization for laser experiments. Subjects underwent sedation using 4 to 6  mg/kg Telazol intramuscularly and anesthesia with 2% to 4% isoflurane delivery. Following intubation with a 4.5- to 5.5-French endotracheal tube, subjects remained on gas anesthesia for the procedure. The veterinary technician placed a catheter (20 g) in an ear vessel to enable venous access in case of an adverse event. Subject monitoring consisted of temperature, respiration, ${\mathrm{SpO}}_{2}$, and heart rate measurements for the duration of the procedure with the use of a SurgiVet monitor (Smiths Medical, Minneapolis, Minnesota). A warm water circulating blanket (Gaymar Inc., Orchard Park, New York) and a Bair Hugger (3M, Maplewood, Minnesota) warm air circulating system maintained the subject’s temperature, in addition to fleece blankets covering unexposed body regions.

The majority of subjects underwent flank shaving with a #50 blade electric clipper (Andis, Sturtevant, Wisconsin), with a select few undergoing waxing with over the counter wax (Nad’s, Austin, Texas) in order to make comparisons relating to the presence of hair follicles. After removing subject flank hair, the veterinary technician gently washed the skin to remove extraneous debris (fecal matter, loose hair, dead skin cells, etc.) and used a black sharpie to draw a grid on the flank surface. Letters and numbers were the labels for the Y and X axes, respectively, to ensure an identifiable location for the placement of laser exposures. Once the experiment had concluded, the subject transferred back to the prep lab for recovery and return to the housing room. During 24-h reads of laser lesions, subjects underwent sedation using 4 to 6  mg/kg Telazol and a mask of isoflurane gas for the length of the procedure. Monitoring of the subject occurred in the same manner as previously described, but intubation and catheter placement was absent due to the short duration of the procedure.

### 2.3 Experimental Protocol

We chose three laser TOTs of 0.01, 0.1, and 10 s as exposure conditions for this experiment. We did not include a TOT of 1 s, as this would have substantially expanded the study size and we believed that the effects of pulse-to-pulse thermal additivity would be similar to the 10-s TOT case. Each TOT set consisted of ten exposures, specifically a single-pulse exposure and nine multiple-pulse exposures. The multiple-pulse exposure trains comprised of 10, 30, or 100 pulses of equal duration, structured at three different duty cycles: 50%, 25%, and 10% for 0.01- and 0.1-s TOT and 80%, 50%, and 25% for 10-s TOT. Shaved porcine skin *in vivo* served as the medium of exposure for all 30 parameter sets. Single-pulses of 0.01-, 0.1-, and 10-s TOT were also exposed on waxed porcine skin to gauge the effect of hair follicle presence on the damage threshold. In total, this study produced 33 damage thresholds associated with a unique laser parameter set. Each set of pulse trains investigated had data from at least three different porcine subject flanks to account for biological diversity.

**Fig. 2:**
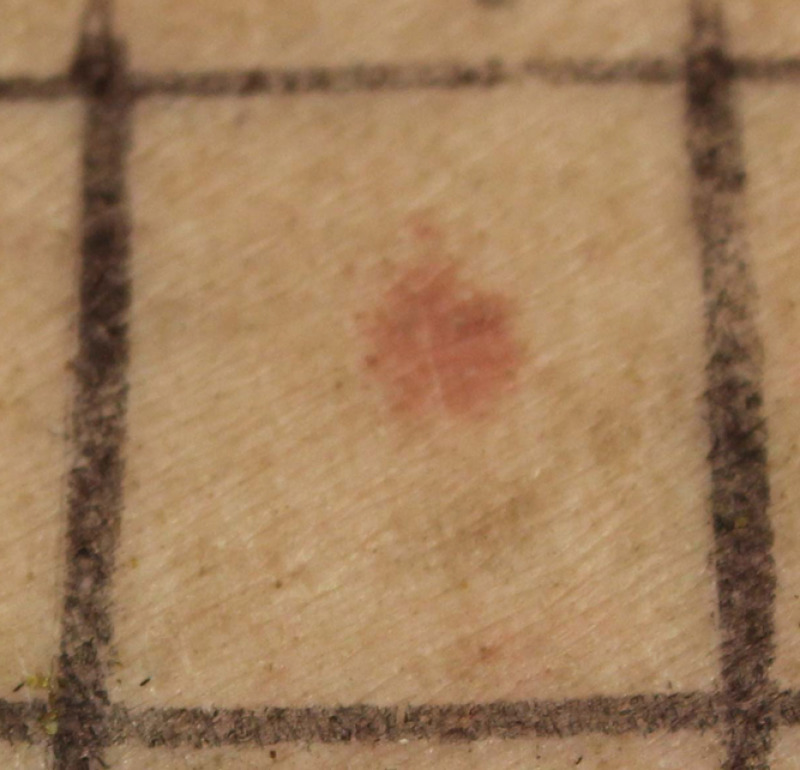
Example of a thermal lesion observed on the skin. This lesion was observed 24 h following exposure to 600 W for 0.1 s from a 1070-nm laser with a 1.04-cm diameter, for a radiant exposure over the $1/e^{2}$ diameter of $71.0\;{J/\mathrm{cm}}^{2}$.

Three experienced observers inspected each exposure site at 1 and 24 h after irradiation and judged the presence or absence of an MVL. Figure 2 depicts an example of a thermal lesion 24-h postexposure. The definition of an MVL was a persistent redness (erythema) on the skin exposure site from laser radiation. A plurality of the three observers determined the end result for each site at the 1- and 24-h intervals following exposure.

### 2.4 Thermal Image Processing

The presence of hair follicles and subject breathing complicated attempts to extract time–temperature histories from the thermal videos. Hair follicles exposed to high irradiance can incinerate and cause smoke and fire, which will also generate “hot spots” on the thermal imagery. These features are more prominent for the shorter-exposure cases with higher irradiance. Subject breathing results in the periodic movement of the heated region away from the specified image point of extraction. This is very evident for longer-exposure cases. We accounted for these effects by developing a Python program to track the center of the heated region, avoid hot spots, and automatically extract time–temperature histories.

For each frame of a thermal video, the program creates an image mask of pixels greater than one standard deviation above the mean (the “exposure mask”), performs a center of mass calculation on the mask, centers a 5×5  pixel region of ROI at the center of mass pixel, and reports the mean value of the ROI as the temperature for that frame. This method allows for tracking of the center of the exposed area as it moves within the thermal camera frame due to subject breathing motion, resulting in a more continuous time–temperature profile. The image processing program then identifies hot spots as pixels within the exposure mask that are greater than two standard deviations above its mean. If the extraction ROI intersects with one of these pixels, the program radially searches outward until the ROI is out of the hot spot for that particular frame.

**Fig. 3:**
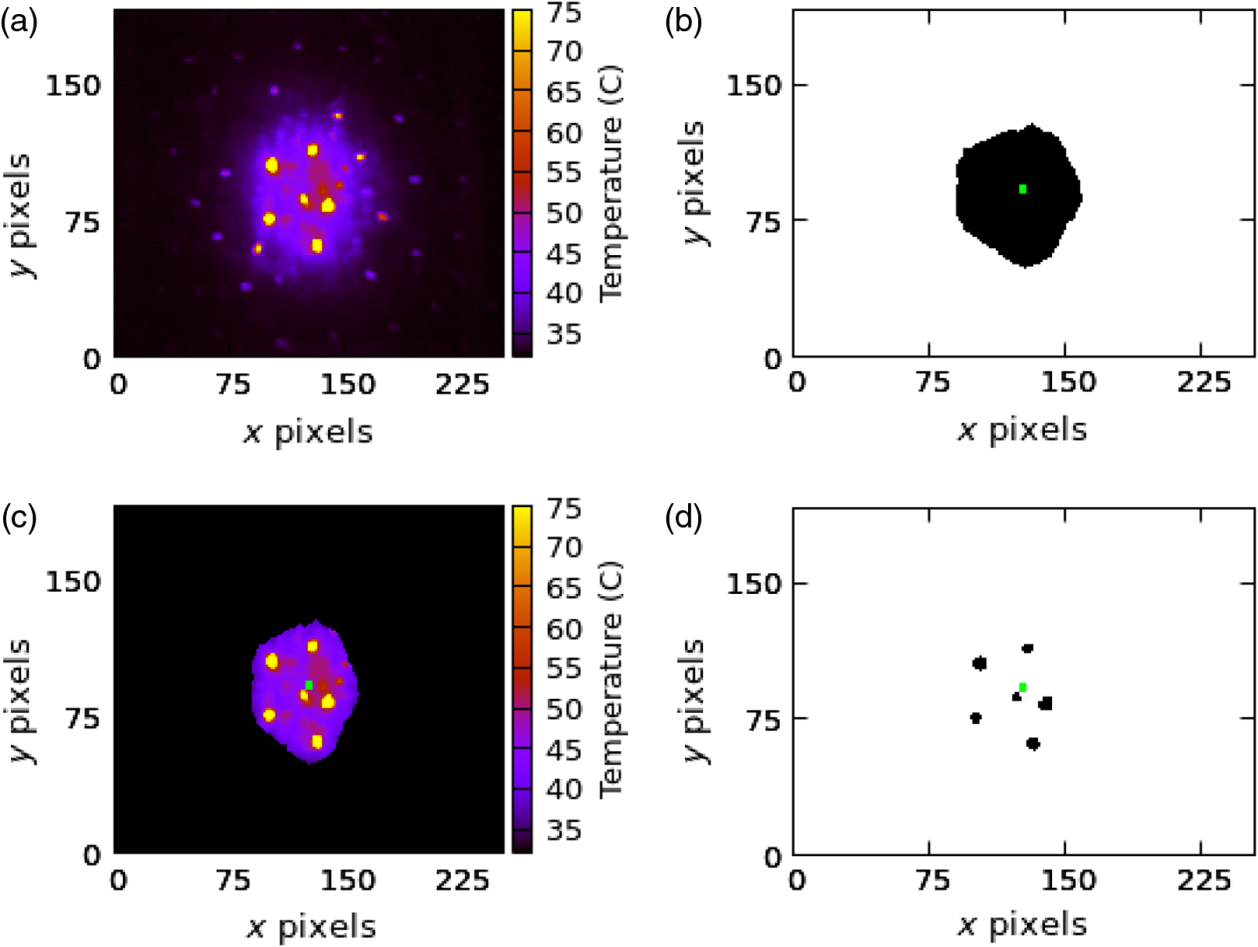
(a) A thermal frame from an exposure and (b) the corresponding binary image mask of the exposure. The highlighted square indicates the ROI around the center of mass of the binary image: (c) the result of multiplying mask and the thermal frame and (d) hair follicles identified within the ROI.

Figure 3(a) shows thermal frame from a 0.01-s exposure with evident hot spots from hair follicles. Figure 3(b) shows the exposure mask where the highlighted square indicates the 5×5  pixel ROI (∼0.64×0.64  mm) at the center of mass. Figure 3(c) shows the resulting masked image from multiplying the binary and thermal image together. Figure 3(d) shows the hot spots in Fig. 3(c) as identified by the algorithm. This thermal extraction technique provides a rapid method for extracting time–temperature profiles and a consistent way to determine peak skin surface temperature during laser exposure with minimal influence from hair follicles.

## 3 Results

### 3.1 Thermal Data Analysis

Although the peak temperatures for exposures within a particular parameter set should theoretically linearly rise with dose, the collected data demonstrated a high degree of noise. This result fits expectations given biological variability and surface tissue inhomogeneity in the form of local absorber distribution. The automated temperature extraction routine was often able to avoid pixels around local absorbers, but it would occasionally fail or simply be unable to evade the effects of hair follicle incineration events, such as flame and smoke. Furthermore, the process of steering the thermal video extraction ROI away from hot spots inevitably distorts the dose–temperature relationship given that the laser beam profile was Gaussian, and thus dose is spatially dependent. If it is desirable to correlate a peak temperature with a statistically generated damage threshold, an intermediate processing step must eliminate probable outliers before fitting the data to a linear response.

For each parameter set, the temperatures (°C) at 10 frames after the laser-off time (+10f) and the associated laser irradiance ($W/{\mathrm{cm}}^{2}$) were fit to a line with the constraint of a y intercept of 32°C, which is a reasonable value for the baseline surface temperature of live porcine skin. For 0.01 and 0.1-s TOT exposures (1440 Hz thermal camera), 10 frames constituted 0.007 s, whereas for 10-s TOT exposures (200 Hz thermal camera), 10 frames constituted 0.05 s. We observed that these postexposure times were sufficient for the most extreme effects of hair follicle incineration to fade. All +10f data outside ±1 standard deviation of the residuals of this initial fit was then discarded, and a second linear fit is performed. The peak temperature data within ±2 standard deviations of the difference between the peak data and the second fit of the +10f data were then used for a third and final linear fit.

**Fig. 4:**
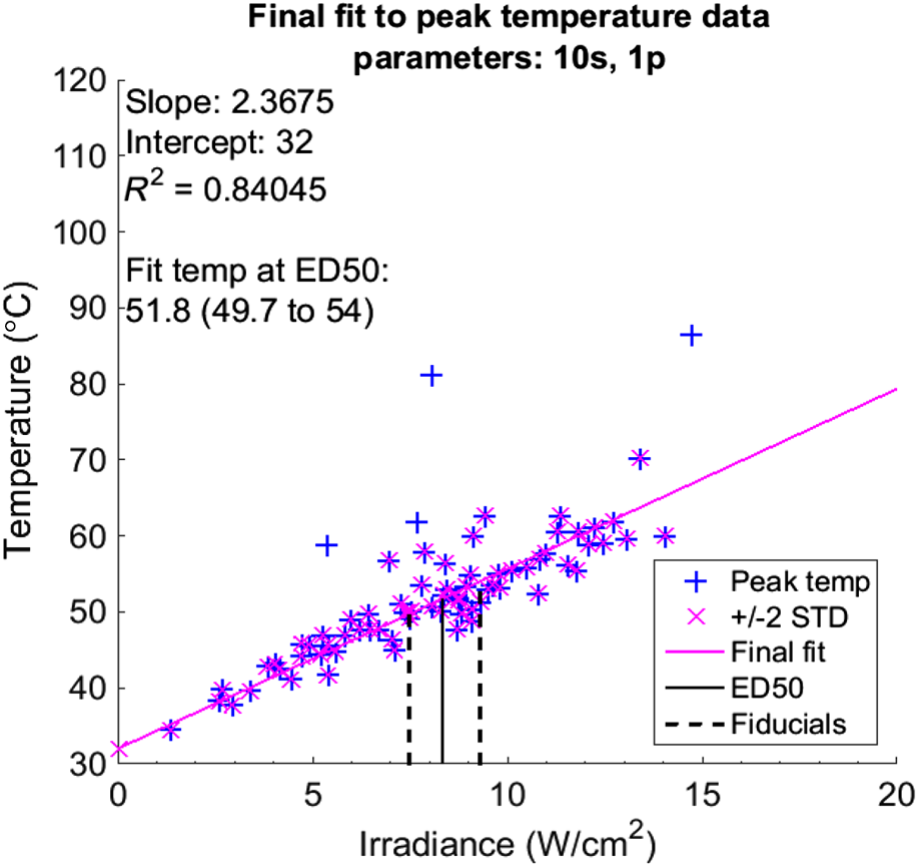
Fit of peak temperature data for a single-laser pulse of 10 s, discarding points outside of ±2  STD of the difference between the peak temperature data and the fitted temperature data at 10 frames after the laser-off time.

**Fig. 5:**
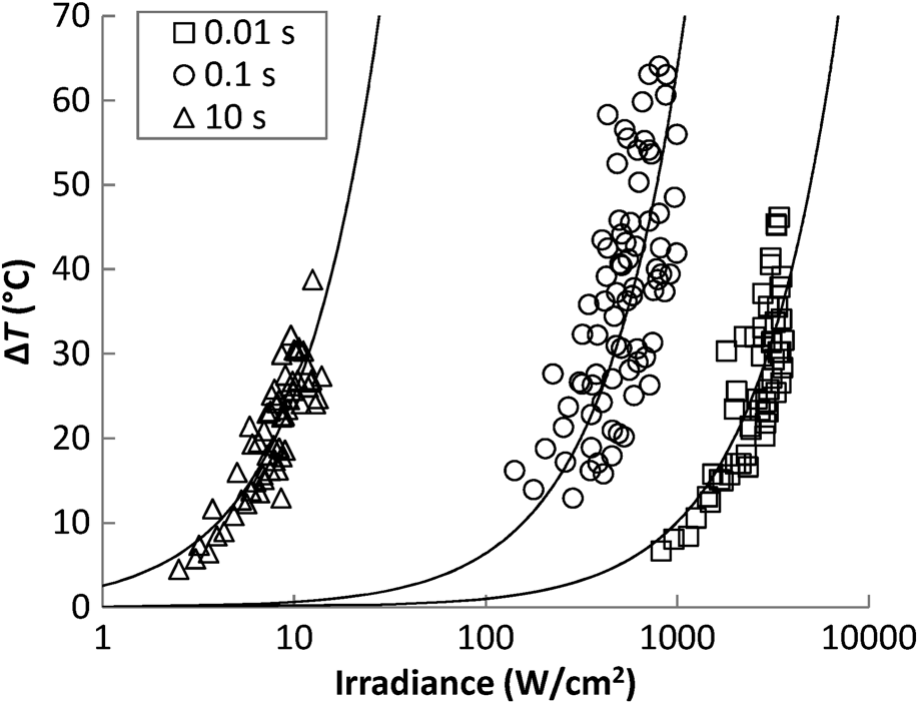
Skin surface temperature changes at all doses for waxed single-pulse exposures of 0.01, 0.1, and 10 s.

The initial fits to the data at 10 frames after laser-off are to bias the final fit away from temperature extractions near local absorbers and burning hair follicles, which will lose heat much faster than the surrounding skin in the moments following exposure. The resulting function can predict the temperature response at any given dose for a set of laser parameters. The extracted peak temperatures and eventual linear fit for single-pulse exposures at 10 s serve as an example in Fig. 4. The plot notes the interception of the fit with the experimentally determined ${\mathrm{ED}}_{50}$ and upper and lower fiducial limits, along with the outlier points excluded from the final fit. Figure 5 exhibits the relationship of irradiance ($W/{\mathrm{cm}}^{2}$) to temperature change in waxed skin for the different exposure durations used in this study.

### 3.2 Probit Analysis

For each parameter set in this study, we used the probit procedure^45^ to estimate the dose threshold for incidence of an MVL. The probit technique calculates the total energy dose required to observe damage in 50% of exposures (${\mathrm{ED}}_{50}$), along with the 95% confidence intervals.

Tables 1–3 display the probit results for laser exposures with 0.01-, 0.1-, and 10-s TOT, respectively. Table 4 provides results for single-pulse laser exposures for both shaved and waxed porcine skin. These tables include the ${\mathrm{ED}}_{50}$ at 1 and 24 h in terms of total radiant exposure ($J/{\mathrm{cm}}^{2}$) along with the lower and upper fiducial limits (95% confidence intervals). These tables also include the fit temperature change (ΔT) at the 24-h ${\mathrm{ED}}_{50}$. The “total sequence duration” column refers to the time duration between the first positive edge and the last negative edge of a pulse train (this value may also be known as the total exposure duration or simply the exposure duration).

**Table 1:** Probit results for lesions 1 and 24 h after exposure to 1070-nm laser pulses with a beam diameter of 1.04 cm ($1/e^{2}$). The laser total on-time for each set of parameters is 0.01 s.

Number of exposures	Number of pulses	Pulse duration (ms)	Duty cycle (%)	PRF (Hz)	Total sequence duration (s)	1-h radiant exposure ED50 and fiducial limits ($J/{\mathrm{cm}}^{2}$)	24-h radiant exposure ED50 and fiducial limits ($J/{\mathrm{cm}}^{2}$)	ΔT (°C)
47	1	10	100	N/A	0.01	14.1 (9.29 to 16.9)	17.8 (15.4 to 19.8)	40.7
46	10	1	50	500	0.019	15.4 (12.8 to 17.1)	17.6 (15.6 to 19.4)	35.2
47	10	1	25	250	0.037	13.0 (5.99 to 16.6)	18.6 (16.0 to 20.7)	33.3
71	10	1	10	100	0.091	20.3 (17.3 to 23.1)	20.0 (17.2 to 22.7)	25.5
46	30	0.333	50	1500	0.0197	15.6 (10.2 to 20.4)	17.2 (14.9 to 19.5)	26.1
69	30	0.333	25	750	0.039	16.2 (13.3 to 18.4)	17.3 (14.9 to 19.3)	24.6
44	30	0.333	10	300	0.097	19.2 (16.8 to 21.4)	19.7 (17.2 to 22.4)	21.9
47	100	0.1	50	5000	0.0199	16.3 (13.7 to 19.4)	15.6 (13.5 to 17.6)	30.0
60	100	0.1	25	2500	0.0397	14.8 (12.2 to 17.1)	16.9 (14.8 to 19.1)	23.7
71	100	0.1	10	1000	0.0991	18.1 (16.1 to 20.5)	18.0 (15.7 to 20.2)	18.1

**Table 2:** Probit results for lesions 1 and 24 h after exposure to 1070-nm laser pulses with a beam diameter of 1.04 cm ($1/e^{2}$). The laser total on-time for each set of parameters is 0.1 s.

Number of exposures	Number of pulses	Pulse duration (ms)	Duty cycle (%)	PRF (Hz)	Total sequence duration (s)	1-h radiant exposure ED50 and fiducial limits ($J/{\mathrm{cm}}^{2}$)	24-h radiant exposure ED50 and fiducial limits ($J/{\mathrm{cm}}^{2}$)	ΔT (°C)
67	1	100	100	N/A	0.1	30.7 (25.0 to 35.6)	33.2 (28.3 to 37.5)	28.6
66	10	10	50	50	0.19	40.5 (35.6 to 45.1)	38.2 (33.7 to 42.5)	25.6
65	10	10	25	25	0.37	40.1 (34.5 to 45.6)	41.6 (36.5 to 46.5)	28.8
68	10	10	10	10	0.91	52.3 (46.5 to 57.7)	52.6 (46.9 to 57.8)	25.7
62	30	3.33	50	150	0.197	39.1 (35.6 to 42.2)	37.4 (32.8 to 41.8)	26.0
61	30	3.33	25	75	0.39	43.0 (38.6 to 47.8)	38.2 (33.9 to 42.2)	27.9
61	30	3.33	10	30	0.97	55.2 (50.3 to 60.7)	49.5 (44.6 to 54.1)	23.4
61	100	1	50	500	0.199	39.0 (34.7 to 43.1)	38.0 (34.5 to 41.4)	27.4
61	100	1	25	250	0.397	45.1 (40.1 to 50.3)	43.7 (39.4 to 48.0)	29.5
61	100	1	10	100	0.991	49.0 (44.4 to 53.9)	48.8 (44.4 to 53.4)	22.6

**Table 3:** Probit results for lesions 1 and 24 h after exposure to 1070-nm laser pulses with a beam diameter of 0.973 cm ($1/e^{2}$). The laser total on-time for each set of parameters is 10 s.

Number of exposures	Number of pulses	Pulse duration (s)	Duty cycle (%)	PRF (Hz)	Total sequence duration (s)	1-h radiant exposure ED50 and fiducial limits ($J/{\mathrm{cm}}^{2}$)	24-h radiant exposure ED50 and fiducial limits ($J/{\mathrm{cm}}^{2}$)	ΔT (°C)
73	1	10	100	N/A	10	69.4 (62.7 to 75.0)	83.7 (75.0 to 93.2)	19.8
50	10	1	80	0.8	12.25	67.9 (60.1 to 73.6)	86.6 (78.3 to 95.4)	20.2
45	10	1	50	0.5	19	86.2 (76.0 to 95.9)	94.8 (86.7 to 104)	19.1
52	10	1	25	0.25	37	95.2 (81.2 to 107)	126 (112 to 145)	19.6
50	30	0.333	80	2.4	12.42	65.9 (39.5 to 77.9)	79.9 (68.9 to 88.5)	20.1
49	30	0.333	50	1.5	19.67	75.6 (64.0 to 82.7)	97.4 (86.1 to 108)	20.1
48	30	0.333	25	0.75	39	100 (88.6 to 109)	120 (109 to 131)	19.1
49	100	0.1	80	8	12.48	68.5 (59.6 to 74.0)	77.9 (69.3 to 85.1)	19.7
40	100	0.1	50	5	19.9	70.5 (50.8 to 83.9)	100 (86.6 to 113)	20.4
54	100	0.1	25	2.5	39.7	100 (86.3 to 111)	121 (105 to 135)	17.5

**Table 4:** Probit results for lesions 1 and 24 h after exposure to a single 1070-nm laser pulse, for shaved and waxed skin.

Waxed or shaved?	Number of exposures	Pulse duration (s)	1-h radiant exposure ED50 and fiducial limits ($J/{\mathrm{cm}}^{2}$)	24-h radiant exposure ED50 and fiducial limits ($J/{\mathrm{cm}}^{2}$)	ΔT (°C)
Shaved	47	0.01	14.1 (9.29 to 16.9)	17.8 (15.4 to 19.8)	40.7
Shaved	67	0.1	30.7 (25.0 to 35.6)	33.2 (28.3 to 37.5)	28.6
Shaved	73	10	69.4 (62.7 to 75.0)	83.7 (75.0 to 93.2)	19.8
Waxed	62	0.01	35.2 (30.5 to 146)	27.2 (25.3 to 28.8)	30
Waxed	84	0.1	40.7 (35.8 to 45.0)	40.8 (36.0 to 44.9)	28.3
Waxed	60	10	75.9 (66.2 to 82.8)	85.9 (79.3 to 92.0)	20

Note that the irradiance ($W/{\mathrm{cm}}^{2}$) or radiant exposure ($J/{\mathrm{cm}}^{2}$) reported in this paper is calculated as the laser power or delivered energy, respectively, divided by the $1/e^{2}$ beam spot area. This approach is similar to that employed by Chen et al.^17^ We do not provide or reference the peak irradiance or peak radiant exposure values, which are determined using the 1/e beam diameter. Given that the 1/e diameter for a Gaussian beam is smaller than the $1/e^{2}$ diameter by a factor of √2, the peak irradiance and radiant exposures are simply twice the reported values.

## 4 Discussion

**Fig. 6:**
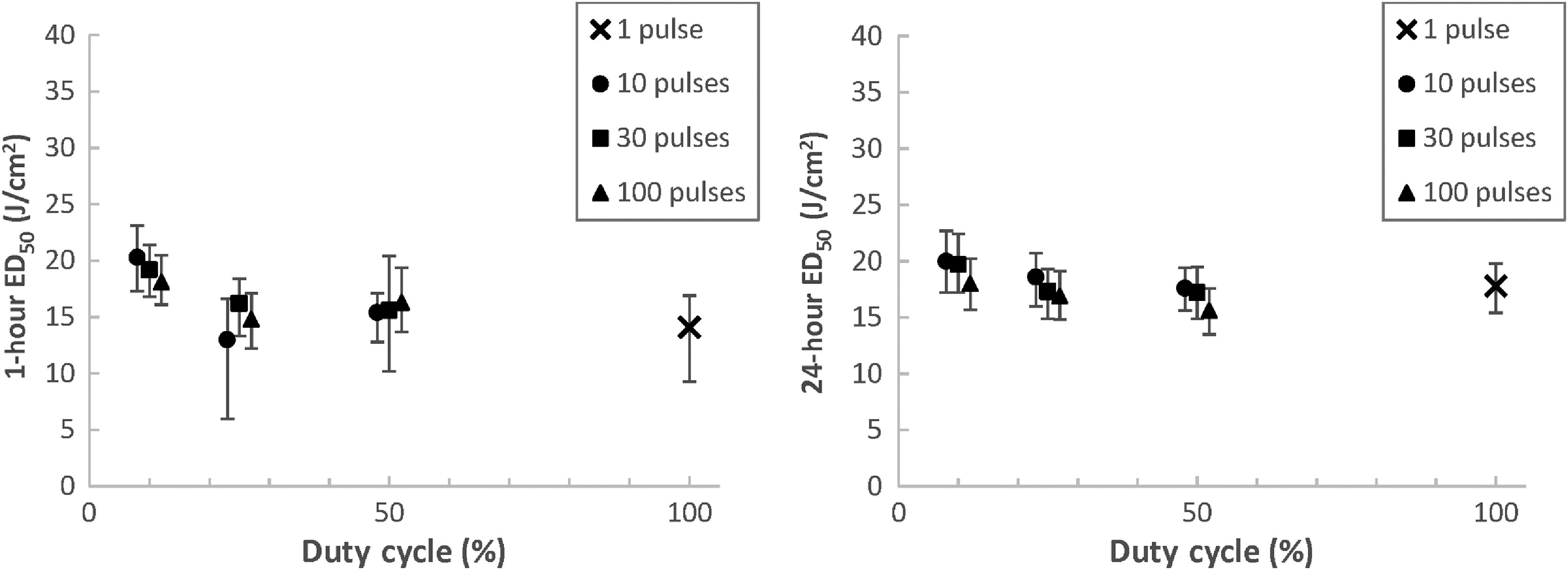
${\mathrm{ED}}_{50}$ values and fiducial limits for shaved porcine skin exposures with 0.01-s TOT.

**Fig. 7:**
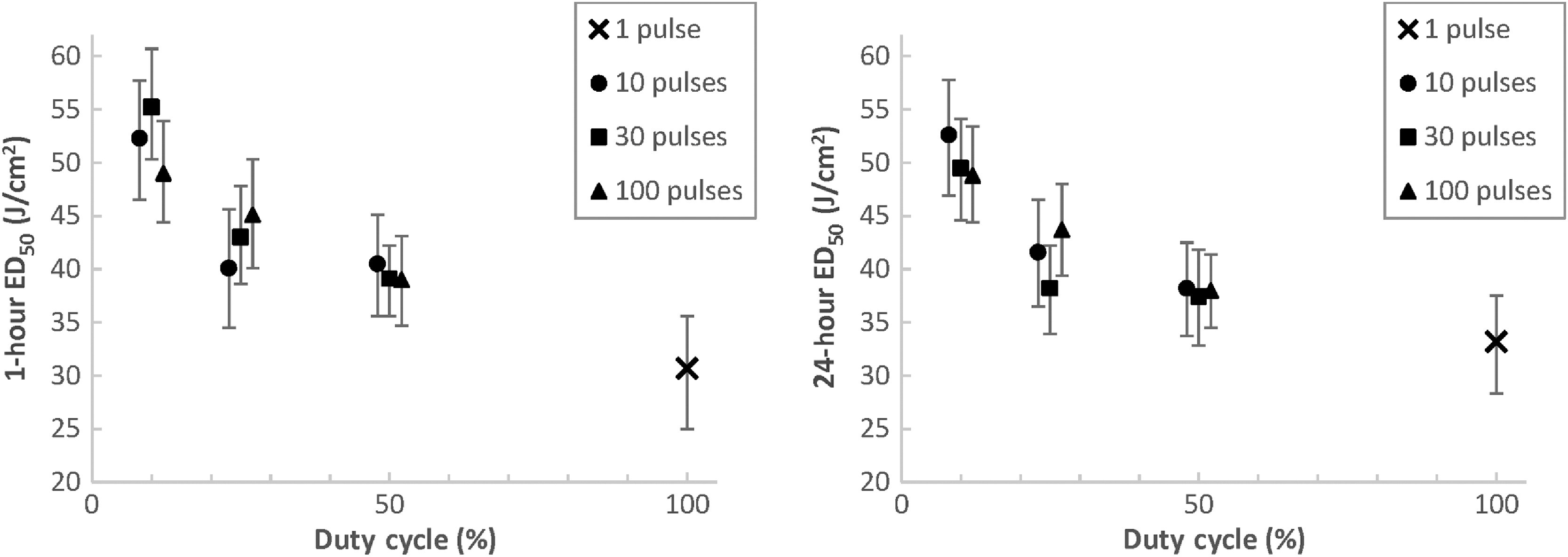
${\mathrm{ED}}_{50}$ values and fiducial limits for shaved porcine skin exposures with 0.1-s TOT.

**Fig. 8:**
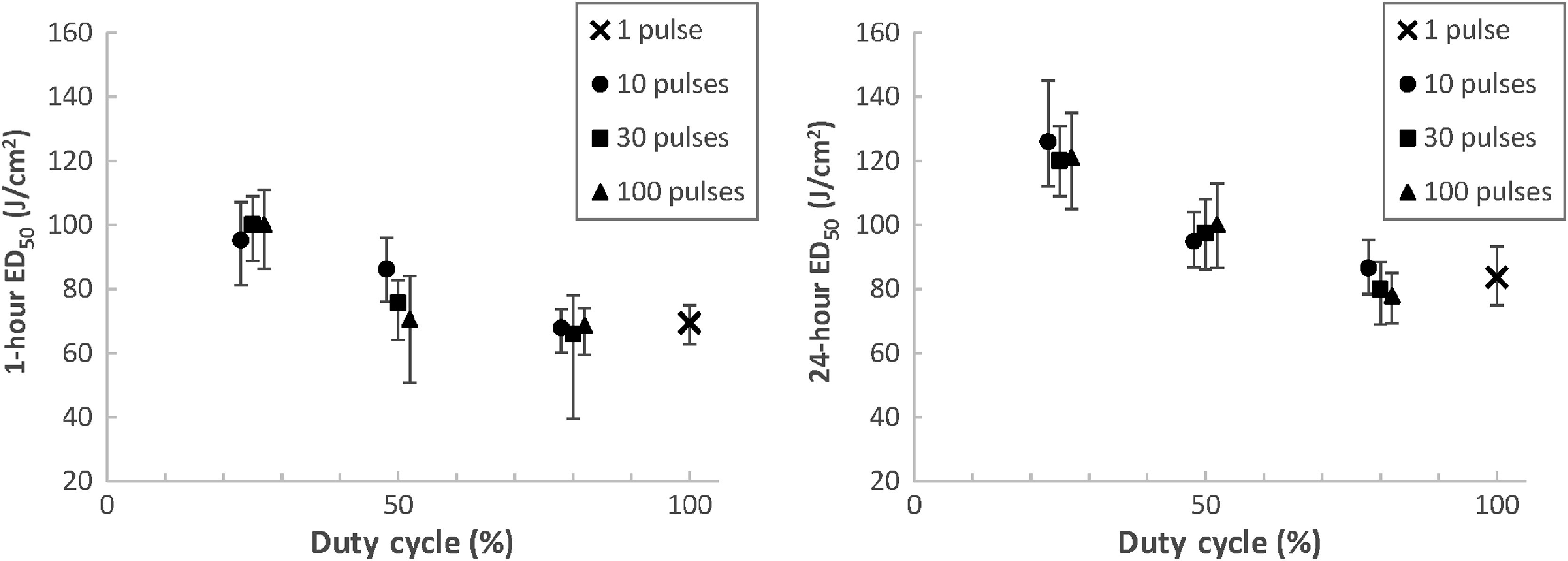
${\mathrm{ED}}_{50}$ values and fiducial limits for shaved porcine skin exposures with 10-s TOT.

Figures 6–8 contain a plot of the calculated 1- and 24-h ${\mathrm{ED}}_{50}$ points bounded by their lower and upper fiducial limits for the shaved porcine skin cases with 0.01-, 0.1-, and 10-s TOT, respectively. The ${\mathrm{ED}}_{50}$ dose is in terms of laser radiant exposure ($J/{\mathrm{cm}}^{2}$), and the data are plotted across duty cycle for variable numbers of pulses.

Figure 6 shows that the ${\mathrm{ED}}_{50}$ and fiducial limits for the 0.01-s TOT cases slightly increases as the duty cycle decreases, for both the 1- and 24-h assessment points. This is understandable given that there was a longer period of laser-off time between pulses at low duty cycles, allowing for heat to diffuse away and the tissue to cool; as such, more total energy was required to reach the point of threshold tissue damage. A notable exception is the 1-h ${\mathrm{ED}}_{50}$ for the 10 pulse, 25% duty cycle case, though the wide fiducial limit range reflects the uncertainty in this particular threshold calculation.

Figure 7 plots the ${\mathrm{ED}}_{50}$ values for the 0.1-s TOT cases. Similar to Fig. 6, the ${\mathrm{ED}}_{50}$ and fiducial limits at both 1- and 24-h increase as duty cycle decreases for a given number of pulses. This trend is even stronger for the 0.1-s TOT case, as there is noticeably less overlap of fiducial limits between cases with different duty cycles when compared to Fig. 6. The same observations are true for the 10-s TOT cases plotted in Fig. 8. Fiducial overlap between different duty cycles is again less prominent than in Fig. 6 but still evident in some cases.

Figure 6 also demonstrates a minor decrease in the 24-h ${\mathrm{ED}}_{50}$ for 0.01-s TOT cases as the total number of pulses increases for a given duty cycle. We know that for pulse trains with the same duty cycles, increasing the number of pulses (which also decreases individual pulse duration) serves to slightly increase the total sequence duration. If there is a high degree of thermal additivity between these pulses, as is the case at high PRFs, we would expect that less total radiant exposure is required to reach an MVL condition when compared to a shorter total sequence duration. This trend is not consistently evident across all duty cycles in Fig. 7 for 0.1-s TOT cases and Fig. 8 for 10-s TOT cases, where there is less thermal additivity between pulses due to longer interpulse laser-off time. However, even for the 0.01-s TOT cases, the effect is relatively minor compared to the ${\mathrm{ED}}_{50}$ calculation noise, as indicated by the degree of change with respect to the fiducial limits.

The difference between the 1-h ${\mathrm{ED}}_{50}$ values and 24-h ${\mathrm{ED}}_{50}$ values is not consistent among the three TOTs. For 0.01-s TOTs, the 1-h ${\mathrm{ED}}_{50}s$ are on average 8.7%±11% less than the 24-h ${\mathrm{ED}}_{50}s$. Of the ten calculated parameter sets, only three feature 1-h thresholds that are greater than the 24-h thresholds. This is in contrast to the 0.1-s TOT exposures, where the 1-h ${\mathrm{ED}}_{50}s$ are on average 2.9%±6.2% greater than the 24-h ${\mathrm{ED}}_{50}s$, with seven of the ten parameter sets featuring higher thresholds at 1 h than 24 h. However, the greatest difference is for the 10-s TOT exposures, where the 1-h ${\mathrm{ED}}_{50}s$ are on average 19%±5.9% lower than the 24-h ${\mathrm{ED}}_{50}s$. In these cases, every 1-h threshold is less than the corresponding 24-h threshold.

The relationships between ${\mathrm{ED}}_{50}s$ for the same parameter set at 1- and 24-h assessment points are not clear in the skin literature. Oliver et al.^12^ observed that for CW exposures to 1319-nm laser radiation for 0.25, 1, 2.5, and 10 s at two different beam diameters, only the 0.25-s cases featured a 1-h ${\mathrm{ED}}_{50}$ that was greater than its 24-h counterpart. However, Vincelette et al.^8^ found that the 1-h ${\mathrm{ED}}_{50}$ was greater than the 24-h ${\mathrm{ED}}_{50}$ in 13 of 16 cases for 1070-nm laser radiation, across a wide range of exposure durations and beam diameters. The three exception cases did not feature the same exposure duration. Regardless of the absolute differences observed, the 1- and 24-h thresholds featured substantial overlapping confidence intervals in both studies. Our data features similar overlapping confidence intervals for the 0.01- and 0.1-s TOT cases, but the relationship for the 10-s TOT cases is more certain, as evidenced by the graphs in Fig. 8 and the data in Table 3. We believe that the particularly long total sequence durations featured in the 10-s TOT data resulted in a greater persistence of erythema following laser exposure.

**Fig. 9:**
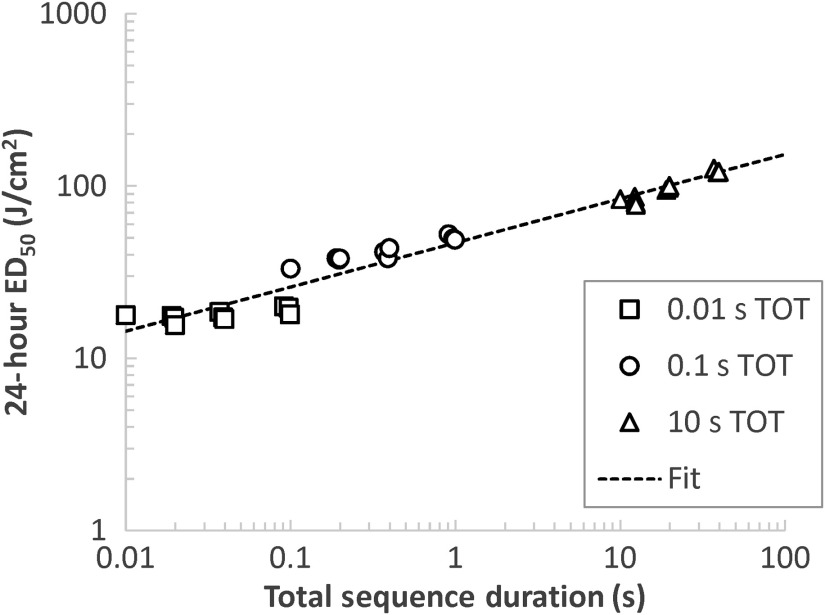
24-h ED50 with respect to total sequence duration for 0.01-, 0.1-, and 10-s TOTs. The dashed line indicates a power function fit to all of the data.

We have noted the effects of duty cycle and number of pulses on the ${\mathrm{ED}}_{50}$ for comparable TOT cases, but the simplest relationship evident in the study is that of the thresholds to the total sequence duration time as seen in Fig. 9. This figure demonstrates the spread of the nonwaxed 24-h ${\mathrm{ED}}_{50}$ values for each TOT across time, with each featuring small clusters of three thresholds taken at the same duty cycle. All of the data falls roughly along the same line in log–log space, which corresponds to a power function. In this case, the data fit to the function:

$$H_{e}=46.9t^{0.257}\;(R^{2}=0.954),$$

where $H_{e}$ is the 24-h radiant exposure ${\mathrm{ED}}_{50}$ ($J/{\mathrm{cm}}^{2}$) and t is the total sequence duration (s). Although this simplification ignores the contributions of the pulse sequence parameters, it recognizes that the photothermal nature of the damage allows for a reasonable prediction of the total radiant exposure threshold based solely the time between the beginning and end of the exposure.

**Fig. 10:**
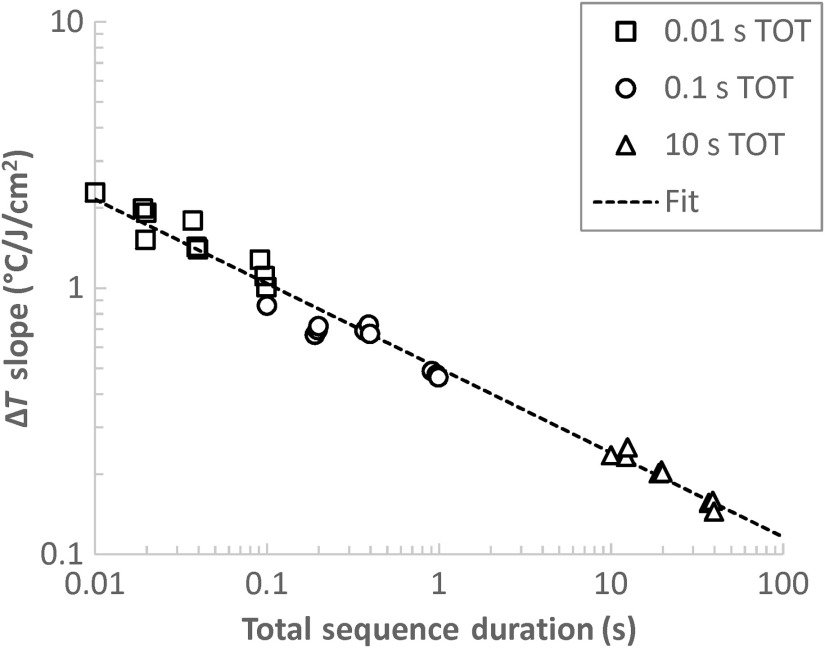
ΔT slope at the 24-h ${\mathrm{ED}}_{50}$ with respect to the total sequence duration. The ΔT slope is the change in peak skin surface temperature at the 24-h ${\mathrm{ED}}_{50}$ over the 24-h radiant exposure ${\mathrm{ED}}_{50}$.

The total sequence duration is also a good indicator of the temperature response at the ${\mathrm{ED}}_{50}$. The surface skin temperature change at the ${\mathrm{ED}}_{50}$, as determined by the technique explained in Sec. 3.1 and listed in the tables in Sec. 3.2, is generally larger for short-duration exposures compared to longer ones. In order to normalize this relationship across all the thresholds, we generate a thermal slope term by dividing the temperature change by the radiant exposure necessary to produce it. Figure 10 displays this slope with respect to the total sequence duration for all of the nonwaxed data in this study. This graph effectively states that the temperature change per unit of radiant exposure at the MVL threshold is inversely proportional to the total sequence duration. The relationship also fits a power function, specifically:

$$m_{T}=0.501t^{-0.317}\;(R^{2}=0.984),$$

where $m_{T}$ is the aforementioned thermal slope term (${}^{\circ}C/J/{\mathrm{cm}}^{2}$) and t is the total sequence duration (s). This finding is compatible with the observations made by Vincelette et al.^8^ and Oliver et al.,^12^ who noted lower peak temperature changes at longer exposure duration ${\mathrm{ED}}_{50}s$ compared to shorter exposure duration ${\mathrm{ED}}_{50}s$.

**Fig. 11:**
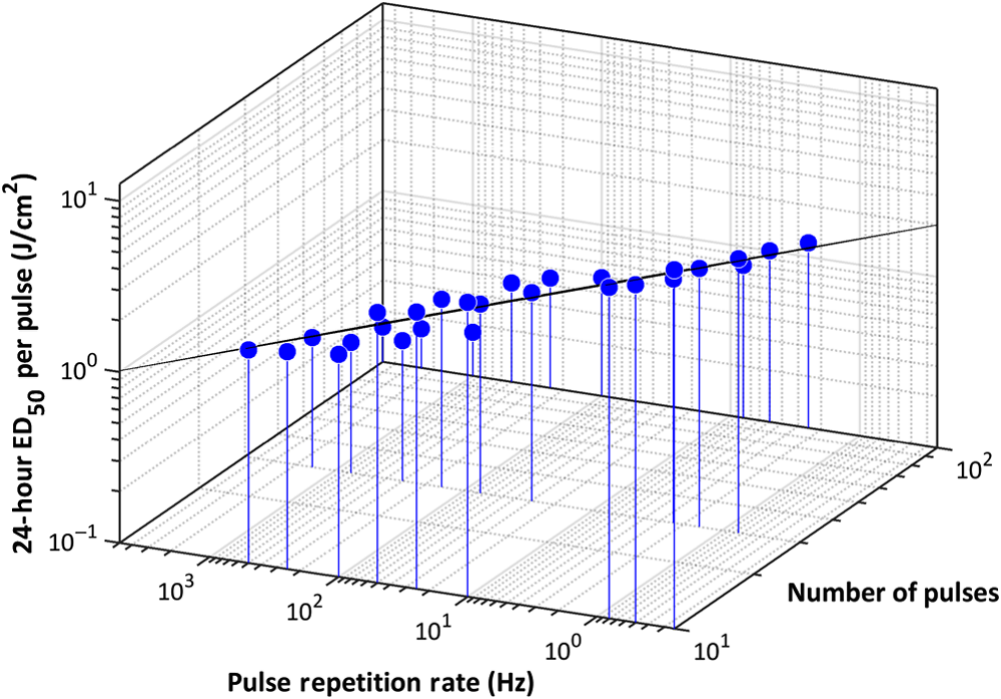
24-h ${\mathrm{ED}}_{50}$ per pulse with respect to the number of pulses and the PRF. The perspective of this plot is on the same plane as the fit to the data.

In studies of multiple-pulse exposures in the eye, it is common to present the energy threshold per pulse as a function of the number of pulses in the train.^23^^,^^30^^–^^32^ This approach is useful for visualizing the cumulative effect of pulses but assumes pulses of equal length and pulse trains with a consistent PRF. These parameters are more diverse in our study given that we held TOT constant and varied the pulse duration and PRF to fit the specified duty cycle and number of pulses. However, it is possible to relate the dose threshold per pulse to the PRF and number of pulses by plotting the data in three dimensions, which can be seen in Fig. 11. Note that the single-pulse exposures at 0.01, 0.1, and 10 s are not included on this plot, as they do not have a defined PRF. Figure 11 demonstrates that the pulsed data are distributed in a plane in this three-dimensional space. We applied a nonlinear least squares technique to fit the data to the function:

$$H_{e,p}=51.0n^{-0.778}f^{-0.250}\;(R^{2}=0.991),$$

where $H_{e,p}$ is the 24-h radiant exposure ${\mathrm{ED}}_{50}$ per pulse ($J/{\mathrm{cm}}^{2}$), n is the number of pulses in the pulse train, and f is the PRF (Hz). The high goodness of the fit according to the $R^{2}$ metric supports the capability to approximate the expected threshold per pulse of a given pulse train with a known number of pulses and PRF, at least in this time region.

Although waxing of the skin will remove most hair follicles and eliminate their transient heating contribution, this is an unrealistic scenario for practical application of MVL study results, as hair is an intrinsic component of many types of skin, especially human skin. It is for this reason that the majority of data points collected in this study (30 primary parameter sets) involved shaved porcine subjects, where the hair follicle remains embedded in this skin. However, the study also investigated three single-pulse cases at 0.01-, 0.1-, and 10-s TOT using waxed porcine subjects. Both shaving and waxing treatments will also change the epidermal surface by removing a portion of the upper stratum corneum layer.

**Fig. 12:**
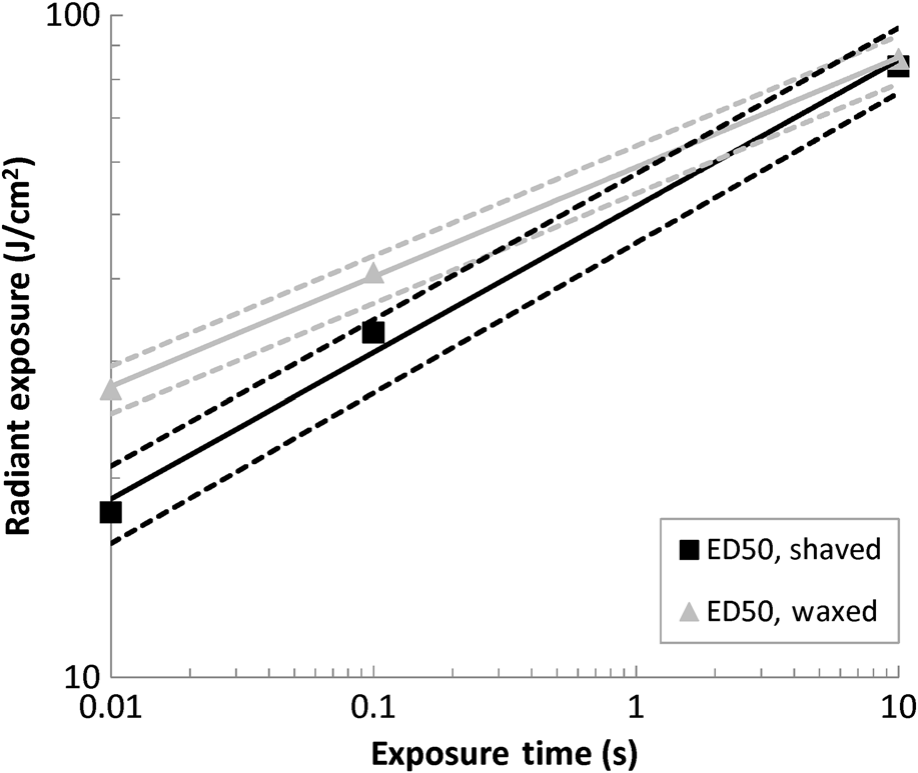
Comparison of the radiant exposure ${\mathrm{ED}}_{50}$ values of waxed and shaved subjects for single-pulse exposures. The dashed lines represent the boundaries of the upper and lower fiducial limits for each ${\mathrm{ED}}_{50}$.

Figure 12 displays plots of the calculated 24-h ${\mathrm{ED}}_{50}$ values for radiant exposure ($J/{\mathrm{cm}}^{2}$) for the single-pulse waxed and shaved cases. This figure represents the fiducial limits for each ${\mathrm{ED}}_{50}$ as dashed lines in order to give an indication of the integrity of the threshold determination. There is evident differentiation between the ${\mathrm{ED}}_{50}$ for shaved subjects and the ${\mathrm{ED}}_{50}$ for waxed subjects for the 0.01- and 0.1-s exposures. In each of these cases, the waxed ${\mathrm{ED}}_{50}$ is greater than the shaved ${\mathrm{ED}}_{50}$, indicating a higher required energy dose to damage waxed tissue. This distinction is very certain, as there is no overlap between the fiducial limits of the two at each exposure time. The presence of hair follicles is less significant at the 10-s exposure time. While the waxed ${\mathrm{ED}}_{50}$ is still slightly greater than the shaved ${\mathrm{ED}}_{50}$, they are very similar and the fiducial limits substantially overlap.

Throughout the course of the study, we observed that the 10-s TOT exposures did not feature nearly as many hair follicle incineration events as the 0.01- and 0.1-s TOT exposures. We believe that this could be due to an irradiance dependence on hair follicle incineration, with most of the 10-s TOT exposure conditions being low enough to allow the follicle to hold and dissipate heat without combusting. At higher irradiances, the hair follicles were more prone to incinerate, scorching the immediately neighboring tissue throughout the shaft and leaving behind carbonizing remains with high optical absorption, which will in turn continue to excessively heat the surrounding tissue throughout the exposure duration until they fully ablate.

**Fig. 13:**
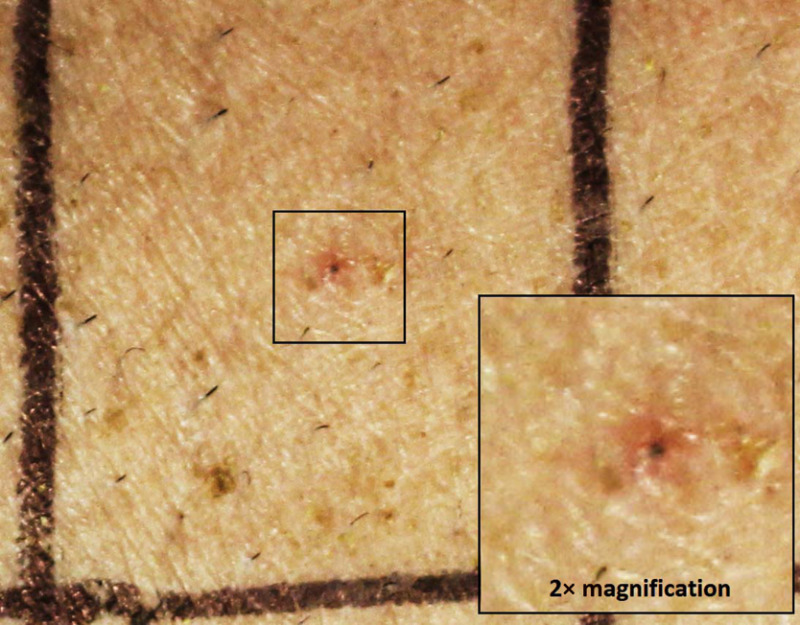
Skin lesion focused around a hair follicle due to a single pulse of 0.01 s and a radiant exposure of $17.6\;{J/\mathrm{cm}}^{2}$.

Hair follicle incineration did not always result in an MVL, but we believe that the process was sufficient to mediate a lower ${\mathrm{ED}}_{50}$ when compared to the hairless case. We have provided an example in Fig. 13. This figure displays minor but visible erythema localized around a partially burned hair follicle, which all three observers judged to be a lesion at the 24-h assessment time. This particular exposure was a single pulse for 0.01 s with a radiant exposure of $17.6\;{J/\mathrm{cm}}^{2}$, a parameter set that Table 4 indicates has a 24-h ${\mathrm{ED}}_{50}$ of $17.8\;{J/\mathrm{cm}}^{2}$ for shaved subjects and $27.2\;{J/\mathrm{cm}}^{2}$ for waxed subjects. In this case, it reasonable to believe that the hair follicle is a significant factor in the presence of erythema.

## 5 Conclusion

This study presents a diverse dataset for porcine skin MVL threshold estimates generated for multiple-pulse laser exposures at 1070-nm using three constant laser on-times (0.01, 0.1, and 10 s) and a beam diameter of ∼1  cm. These results span a variety of individual pulse durations, numbers of pulses, duty cycles, pulse repetition frequencies, and total sequence durations, for a total of 30 unique parameter sets for porcine skin *in vivo*. We have mapped the experimentally determined radiant exposure threshold per pulse to a function of the number of pulses and PRF. Damage in this exposure time region is primarily photothermal and is strongly reliant on the total sequence duration between the onset of the first pulse and conclusion of the last pulse. The presence of hair follicles contributes to a slightly lower threshold for skin damage when compared to exposures on waxed skin, an effect that is more evident at shorter exposure times. The results of these experiments will further inform the standards for safe use of lasers and provide a vast dataset for refinement and validation of efforts to computationally model laser bioeffects.
